# Transplant Prognosis in Kidney Transplant Recipients with Diabetes under Mycophenolic Acid-Focused Therapeutic Drug Monitoring

**DOI:** 10.3390/jpm11111224

**Published:** 2021-11-18

**Authors:** Eisuke Nakamura, Tadashi Sofue, Yasushi Kunisho, Keisuke Onishi, Kazunori Yamaguchi, Emi Ibuki, Rikiya Taoka, Nobufumi Ueda, Mikio Sugimoto, Tetsuo Minamino

**Affiliations:** 1 Division of Nephrology and Dialysis, Department of Cardiorenal and Cerebrovascular Medicine, Faculty of Medicine, Kagawa University, Takamatsu 761-0793, Kagawa, Japan; nakamura.eisuke@kagawa-u.ac.jp (E.N.); kunisho.yasushi@kagawa-u.ac.jp (Y.K.); onishi.keisuke@kagawa-u.ac.jp (K.O.); minamino.tetsuo.gk@kagawa-u.ac.jp (T.M.); 2 Department of Pharmacy, Kagawa University Hospital, Takamatsu 761-0793, Kagawa, Japan; yamaguchi.kazunori@kagawa-u.ac.jp; 3 Department of Pathology, Faculty of Medicine, Kagawa University, Takamatsu 761-0793, Kagawa, Japan; ibuki.emi@kagawa-u.ac.jp; 4 Department of Urology, Faculty of Medicine, Kagawa University, Takamatsu 761-0793, Kagawa, Japan; taoka.rikiya@kagawa-u.ac.jp (R.T.); ueda.nobufumi@kagawa-u.ac.jp (N.U.); sugimoto.mikio@kagawa-u.ac.jp (M.S.)

**Keywords:** kidney transplantation, diabetes, mycophenolic acid, therapeutic drug monitoring

## Abstract

Mycophenolate mofetil is a key immunosuppressant that is metabolized into mycophenolic acid (MPA). The prognostic impact of MPA-focused therapeutic drug monitoring on allograft prognosis has not been determined in kidney transplant recipients with diabetes. In this study, we assessed the pharmacokinetics of MPA and allograft prognosis in recipients with diabetes. This study retrospectively analyzed 64 adult kidney transplant recipients. MPA blood concentration data (e.g., the time to the maximum concentration (Tmax), and the area under the concentration–time curve from 0 to 12 h (AUC_0–12_)) were collected at 3 weeks and 3 months after kidney transplantation. Of the 64 recipients, 15 had pre-existing diabetes. At 3 months after kidney transplantation, the Tmax of MPA was significantly longer in recipients with diabetes (mean (standard deviation): 2.8 (2.1) h) than in recipients without diabetes (1.9 (1.1) h, *p* = 0.02). However, the allograft estimated glomerular filtration rate and acute rejection rate, including borderline change, did not differ according to the diabetes status in patients with adjusted AUC_0–12_ of MPA within the target range. In conclusion, a longer Tmax of MPA was observed in recipients with diabetes; however, acceptable allograft prognosis was observed in kidney transplant recipients with diabetes and a sufficient AUC_0–12_ of MPA.

## 1. Introduction

Diabetic nephropathy is a major complication of type 2 diabetes mellitus and a leading cause of end-stage renal disease [1,2]. Patient and graft survival rates are reportedly similar among recipients regardless of their diabetes status [3,4]. However, patients with diabetic nephropathy often have cardiovascular complications, and they may be hesitant to undergo kidney transplantation as a form of renal replacement therapy [5]. Furthermore, a lower survival rate has been reported among transplant recipients with pre-existing diabetes because of their increased risks of cardiovascular and infectious diseases [6,7]. The maintenance of an appropriate concentration of immunosuppressive agents is important for maximizing patient and graft survival after kidney transplantation, and it helps to minimize the side effects of immunosuppressive agents.

Mycophenolate mofetil (MMF) is a commonly used immunosuppressant that is metabolized to mycophenolic acid (MPA). MMF is rapidly absorbed in the upper gastrointestinal tract, and the MPA blood concentration varies according to concomitant medication use; thus, therapeutic drug monitoring (TDM) is needed [8] to maintain an appropriate area under the blood concentration–time curve (AUC). Previous randomized controlled trials demonstrated that MPA-focused TDM in patients with a kidney transplantation significantly reduced the incidence of acute allograft rejection (AR) compared with fixed-dose MPA [9,10].

In addition to diabetic nephropathy, diabetic gastroenteropathy is a major complication of diabetic autonomic neuropathy that is characterized by the impaired peristalsis of the intestinal tract. Drug absorption is reportedly delayed in patients with diabetic gastroenteropathy because of impaired peristalsis [10]. Furthermore, the time to the maximum concentration (Tmax) of MPA is longer in renal transplant recipients with diabetic gastroparesis than in recipients without diabetes [11,12,13,14]. However, the prognostic impact of MPA-focused TDM on allograft prognosis has not been determined in patients with diabetes.

In this study, we investigated the pharmacokinetics of MPA in kidney transplant recipients with diabetes and examined the allograft prognosis in patients with a sufficient AUC of MPA after MPA-focused TDM.

## 2. Materials and Methods

### 2.1. Patients

We retrospectively analyzed 64 consecutive adults who underwent living-donor kidney transplantation with an MMF-based immunosuppressant regimen (CellCept™, Chugai Pharmaceutical Co. Ltd., Tokyo, Japan) at Kagawa University Hospital, between August 2003 and March 2013. The recipients of deceased-donor kidney transplantation and recipients who did not receive MMF-based regimens were excluded from this study. Informed consent was obtained from each participant in this study. The protocols, patient information, and informed consent forms were reviewed and approved by the Ethics Committee of Kagawa University (#H27-020).

### 2.2. Study Design

The 64 recipients were classified into 2 subgroups according to their diabetes status before transplantation: recipients with pre-existing diabetes mellitus comprised the DM group (*n* = 15) and recipients without diabetes mellitus comprised the non-DM group (*n* = 49). The diagnostic criteria for diabetes were HbA1c ≥ 6.5% or the use of diabetes medications. The risk factors for diabetic autonomic neuropathy were evaluated by the type of diabetes, duration of diabetes, presence of retinopathy, insulin use, and oral medications in the DM group. The recipient immunological risk was assessed by ABO blood type compatibility, human leukocyte antigen (HLA) mismatch, and flow cytotoxic crossmatch tests.

The immunosuppressive regimens involved combinations of four drugs: methylprednisolone, MMF, calcineurin inhibitors (tacrolimus or cyclosporine), and basiliximab. The type of calcineurin inhibitor was not switched during the study. The allograft kidney function was evaluated using the estimated glomerular filtration rate (eGFR), as determined by the Modification of Diet in Renal Disease equation for Japanese patients [15]. Allograft eGFR was evaluated at 3, 12, and 36 months after kidney transplantation. Allograft kidney biopsy samples were obtained from protocol biopsies at 1 and 3 years after kidney transplantation. They were also obtained from episode biopsies. Acute allograft rejection, including borderline change, was defined as the total number of ARs and borderline changes based on the Banff score criteria [16].

### 2.3. Blood MPA Concentration

Blood MPA concentrations were determined by the Viva-E System (Siemens Healthineers, Tokyo, Japan) using the Emit 2000 MPA Assay (Siemens Healthineers, Tokyo, Japan), Emit 2000 Mycophenotic Calibrators (Siemens Healthineers), and Emit 2000 Mycophenotic Control (Siemens Healthineers) [17]. MMF administration was started 7 days before ABO-compatible kidney transplantation and 14 days before ABO-incompatible kidney transplantation. The initial dose of MMF was 2000 mg; however, if the recipient’s body weight was less than 60 kg, then the initial dose of MMF was 1500 mg. MMF was orally administered twice daily at 09:00 and 21:00 (approximately 2 h after meals).

MPA blood concentration data (e.g., trough blood concentration (C0), Tmax, maximum concentration (Cmax), AUC from 0 to 12 h (AUC_0–12_), and dose-normalized AUC) were determined at 3 weeks after kidney transplantation. These data were also collected at 3 and 12 months after kidney transplantation. The C0 of MPA was defined as the blood MPA concentration, measured immediately before MMF administration.

For the AUC analysis, whole-blood samples were collected immediately before oral MPA administration at 09:00, as well as 1, 2, 4, 6, and 8 h after the morning dose. Samples were also collected shortly before the evening dose (i.e., 12 h after the morning dose). AUC_0–12_ was calculated using the linear trapezoidal rule. After AUC analysis, the dose of MMF was adjusted to achieve MPA AUC_0–12_ of 40–70 µg/h/mL. The dose-normalized AUC was calculated using the following calculating formula: MPA dose-normalized AUC = MPA AUC/MPA morning dose × 1000.

### 2.4. Statistical Analysis

All analyses were performed using SPSS Statistics, version 26.0 for Windows (IBM Japan, Tokyo, Japan). Non-normally distributed variables were expressed as the median and interquartile range (IQR), whereas normally distributed variables were expressed as the mean and standard deviation (SD). The Kolmogorov–Smirnov test was used to assess the normality of data distribution. Clinical variables were compared between the groups using the χ^2^ test for categorical variables and the Student’s *t*-test, one-way analysis of variance, or two-way analysis of variance for continuous variables. The log rank analysis was used to examine the rate of post-transplant AR. *p* < 0.05 indicated statistical significance.

## 3. Results

The baseline recipient characteristics stratified according to the diabetes status before transplantation are presented in Table 1. No significant differences were observed in age, sex, body mass index, dialysis vintage, or the morning dose of MMF between the two groups. Furthermore, no significant differences were observed in the doses and C0 of tacrolimus or cyclosporine, or in the doses of prednisolone between the two groups. The HbA1c level was significantly higher in the DM group than in the non-DM group (*p* < 0.01). The number of HLA mismatch was significantly higher in the DM group than in the non-DM group (*p* = 0.04). The rate of positivity in the flow cytotoxic crossmatch tests was similar between the two groups.

The risk factors for diabetic autonomic neuropathy in the DM group are presented in Table 2. Only one patient had type 1 diabetes. The median duration of diabetes was 240 (156–252) months, and most patients had diabetic retinopathy (*n* = 14/15, 93%) and required insulin treatment. Only one patient was receiving dipeptidyl peptidase-4 inhibitor treatment.

The total number of patients who required an MMF dose adjustment after the AUC assessment from 3 weeks to 3 months and from 3 months to 1 year was similar between the DM (13/30, 43%) and non-DM groups (52/98, 53%, *p* = 0.41). 

The Tmax of MPA at 3 weeks after transplantation was significantly longer in the DM group (mean (SD): 3.5 h (2.3)) than in the non-DM group (2.3 (1.4) h, *p* = 0.02). There were no significant differences in morning C0, Cmax, AUC_0–12_, or dose-normalized AUC of the MPA between the two groups (Table 3).

The Tmax of MPA at 3 months after transplantation was significantly longer in the DM group (mean (SD): 2.8 (2.1) h) than in the non-DM group (1.9 (1.1) h, *p* = 0.02). There were no significant differences in morning C0, AUC_0–12_, or dose-normalized AUC between the two groups; however, the Cmax at 3 months after transplantation was significantly lower in the DM group (mean (SD): 8.2 (4.4) mg/dL) than in the non-DM group (11.5 (5.5) mg/dL, *p* = 0.04, Table 4).

**Figure 1:**
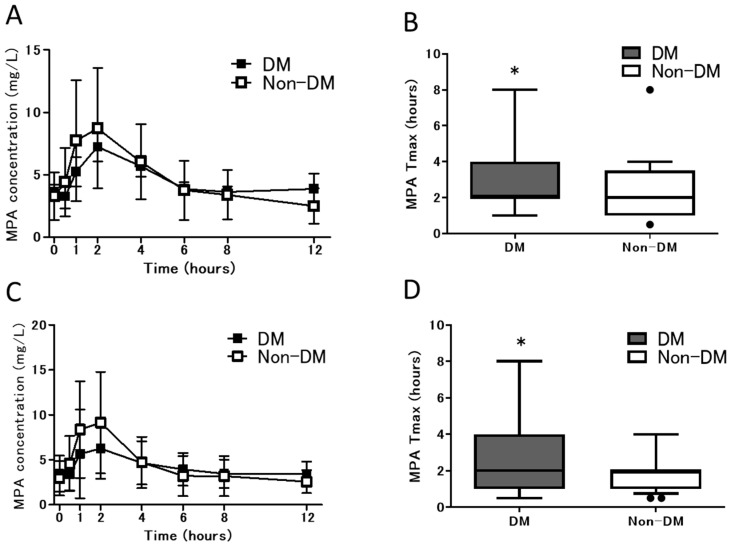
MPA pharmacokinetics at 3 weeks and 3 months in the DM and Non-DM groups. (**A**) Mean concentration–time profile of MPA at 3 weeks, (**B**) Tmax of MPA at 3 weeks, (**C**) mean concentration–time profile of MPA at 3 months, and (**D**) Tmax of MPA at 3 months. Abbreviations: MPA, mycophenolic acid; Tmax, time to maximum concentration; DM, patients with diabetes; and non-DM, patients without diabetes.

MPA pharmacokinetics at 3 weeks and 3 months after transplantation is presented in Figure 1. At 3 weeks and 3 months after transplantation, the Cmax of MPA tended to be lower in the DM group than in the non-DM group, whereas the Tmax of MPA was significantly longer in the DM group (* *p* < 0.05). However, the MPA blood concentration tended to be higher in the DM group after 6 h of administration, whereas the AUC_0–12_ was equivalent between the groups.

**Figure 2:**
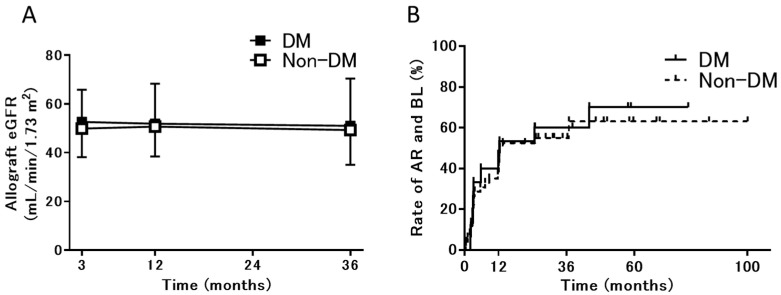
Allograft eGFR and AR incidence in the DM and non-DM groups. (**A**) Allograft eGFR at 3, 12, and 36 months after transplantation and (**B**) the cumulative incidence of AR, including BL. Abbreviations: DM, patients with diabetes; non-DM, patients without diabetes; eGFR, estimated glomerular filtration rate; AR, acute rejection; and BL, borderline change.

The allograft eGFR and AR rate are presented in Figure 2. The allograft eGFR did not differ between the 2 groups from 3 to 36 months after transplantation (Figure 2A), and the AR rate, including the incidence of borderline change, was also similar between the 2 groups (Figure 2B). Most instances of AR involved borderline changes (non-DM group, 27/28; DM group, 8/10; *p* = 0.16) that did not require additional immunosuppressive treatment. BK virus nephropathy, patient death, and graft loss were not observed in either group during the observation period.

## 4. Discussion

In this study, we clarified the pharmacokinetic characteristics of MPA in Japanese kidney transplant recipients with diabetes. Pharmacokinetics analysis of MPA in kidney transplant recipients with diabetes revealed a longer Tmax and lower Cmax compared with the results in recipients without diabetes; however, we could control AUC_0–12_ within the target range among recipients regardless of their diabetes status. We also demonstrated that the rates of AR and graft function were similar in recipients with and without diabetes who achieved an equivalent AUC of MPA. 

Patients with end-stage renal disease caused by diabetes have higher cardiovascular risk and mortality rates than patients with other underlying diseases [18]. Although kidney transplantation reportedly improves patient prognosis, compared with the effects of dialysis [19], there is a need to understand the specific pharmacokinetics of patients with diabetes. A previous study revealed slow drug absorption in recipients with diabetes because of the delayed excretion of gastric contents, which resulted in a longer Tmax of MPA [11,12,14]. It has been reported that the severity of diabetic autonomic neuropathy, including diabetic gastroenteropathy, is associated with the severity of diabetic retinopathy and diabetic nephropathy [20]. Because the patients with diabetes in this study had a longer duration of diabetes and a higher prevalence of retinopathy, we considered them to comprise a population with a higher severity of diabetic autonomic neuropathy, including diabetic gastroenteropathy. In the present study, a significantly longer Tmax of MPA was observed at both 3 weeks and 3 months after transplantation in recipients with pre-existing diabetes. Furthermore, a lower Cmax of MPA was observed in recipients with diabetes at 3 months after transplantation. These results suggest that dose-controlled MMF management for kidney transplant recipients with diabetes can increase the risk of acute rejection. A previous report illustrated that pre-existing diabetes in kidney transplant recipients was an independent risk factor for cellular rejection [21]. In the present study, although the rate of HLA mismatch was higher in the DM group, the rate of AR, including borderline change, was similar between the groups. These data indicated that the diabetic group in this study was not an immunological high-risk group. It is unclear whether diabetes-related delayed drug absorption alone is a risk factor for AR, although it may be a contributing factor. Therefore, MPA-focused TDM is presumably useful, especially in recipients with pre-existing diabetes.

In this study, we performed MPA dose adjustment according to the results of AUC analysis and observed equivalent allograft kidney function and rejection rates in recipients with and without pre-existing diabetes. The AR incidence reportedly decreases when the AUC_0–12_ of MPA is maintained above 30–40 µg/h/mL [22,23,24]. However, the side effect frequency reportedly increases when the AUC of MPA exceeds 60 µg/h/mL [25]. The Emit 2000 method was used in our study to measure MPA blood concentrations. This method produces 10–20% greater values than high-performance liquid chromatography [26]. Accordingly, we controlled the AUC of MPA at approximately 40–70 µg/h/mL. In this study, AUC measurements were performed before transplantation and at 3 weeks and 3 months after transplantation. The results were used to guide dose adjustment to maintain the AUC of MPA within the target range. Therefore, the AUC of MPA in both groups was maintained at approximately 50 µg/h/mL at 3 weeks and 3 months after transplantation. The transplant prognosis did not differ between the groups. These findings indicated that the delayed drug absorption of MPA in patients with diabetes did not have a substantial prognostic impact when a sufficient AUC of MPA was maintained.

There were some limitations in our study because of its small-scale and cross-sectional design. First, the usefulness of TDM-guided controlled dosing compared with fixed dosing could not be confirmed. Second, because the study population was comparatively small and the observation period was short, we believe that the results of this study should be considered as preliminary data. Additional large-scale and long-term observations are needed. Third, the current study design did not allow for comparisons of patients who did and did not undergo TDM in the DM group, and, thus, it was not possible to determine whether a longer Tmax or lower Cmax directly affected the prognosis after transplantation. Fourth, because our study population was not an immunological high-risk group, we included borderline changes as AR. For a more accurate analysis, it may be necessary to evaluate AR as an outcome in high-risk populations. Transplant prognosis is strongly affected by both AUC of MPA and other factors, including the AUC of calcineurin inhibitors and background factors, such as surgery, primary disease, and donor characteristics. Thus, we were unable to evaluate the strength of the relationship between the AUC of MPA and the transplant prognosis. Future evaluations that adjust for these confounding factors in larger populations are needed.

In conclusion, a longer Tmax and lower Cmax of MPA were observed in kidney transplant recipients with diabetes; however, acceptable allograft prognosis was observed in patients with sufficient AUC_0–12_ of MPA under MPA-focused TDM.

## Figures and Tables

**Table 1:** Baseline recipient characteristics stratified by the diabetes status.

	DM	Non-DM	*p*-Value
Recipients, *n*	15	49	
Age, years (SD)	51.1 (13.3)	47.9 (12.2)	0.39
Men, *n* (%)	11 (73%)	36 (73%)	1.00
Body mass index, kg/m^2^ (SD)	22.6 (3.7)	22.4 (4.3)	0.86
Dialysis vintage, months (IQR)	8.5 (4.8–36)	9.0 (0–46)	0.84
HbA1c, % (SD)	5.6 (0.6)	4.9 (0.4)	<0.01 *
MMF morning dose, mg (SD)	696 (107)	690 (108)	0.85
CyA/Tac (*n*)	2/13	6/43	1.00
CyA/Tac daily dose (mg)	175/5.8	203/4.6	0.55/0.19
CyA/Tac trough concentration (µg/L)	129/8.2	205/10.1	0.30/0.74
PSL daily dose (mg)	4.0 (0)	4.1 (0.5)	0.33
ABO-blood type incompatible, *n* (%)	7 (46%)	19 (39%)	0.76
HLA mismatch, *n* (SD)	4.7 (1.35)	3.5 (1.65)	0.04 *
FCXM positive for T-cell, *n* (%)	0/9 (0%)	4/31 (12.9%)	0.56
FCXM positive for B-cell, *n* (%)	4/9 (44.4%)	9/31 (29.0%)	0.44

Continuous variables are described as the median (interquartile range (IQR)) or mean (standard deviation (SD)). Categorical variables are described as *n* (%). Abbreviations: DM, patients with diabetes mellitus; non-DM, patients without diabetes; MMF, mycophenolate mofetil; CyA, cyclosporine; Tac, tacrolimus; PSL, prednisolone; HLA, human leukocyte antigen; and FCXM, flow cytotoxic crossmatch. * *p* < 0.05.

**Table 2:** Risk factors for diabetic autonomic neuropathy in the DM group.

	Results
Type 1 diabetes	1 (7%)
Duration of diabetes, months (IQR)	240 (156–252)
Diabetic retinopathy, *n* (%)	14 (93%)
Use of insulin, *n* (%)	14 (93%)
Use of DPP-4 inhibitor, *n* (%)	1 (7%)
Use of GLP-1 receptor agonist, *n* (%)	0 (0%)

Values represent the median (interquartile range (IQR)) or *n* (%). Abbreviations: DM, patients with diabetes mellitus; DPP4, dipeptidyl peptidase-4; and GLP-1, glucagon-like peptide-1.

**Table 3:** Pharmacokinetic profile of mycophenolic acid at 3 weeks after transplantation.

	DM	Non-DM	*p*-Value
Recipients, *n*	15	49	
Tmax (h)	3.5 (2.3)	2.3 (1.4)	0.02 *
Morning trough levels (mg/L)	3.6 (2.4)	3.3 (1.9)	0.63
Cmax (mg/L)	8.4 (4.2)	10.7 (4.8)	0.11
AUC0–12 (µg/h/mL)	54.8 (25.9)	57.7 (23.7)	0.69
Dose-normalized AUC (mg/h/L) †	79.7 (40.1)	85.6 (37.4)	0.61

Values represent the mean (standard deviation) or *n* (%). Abbreviations: DM, patients with diabetes mellitus; non-DM, patients without diabetes; Tmax, time to maximum concentration; Cmax, maximum concentration; and AUC0–12, area under the concentration–time curve from 0 to 12 h. † Dose-normalized AUC values were normalized to 1000 mg of mycophenolate mofetil. * *p* < 0.05.

**Table 4:** Pharmacokinetic profile of mycophenolic acid at 3 months after transplantation.

	DM	Non-DM	*p*-Value
Recipients, *n*	15	49	
Tmax (h)	2.8 (2.1)	1.9 (1.1)	0.02 *
Morning trough levels (mg/L)	3.5 (2.0)	3.0 (1.9)	0.39
Cmax (mg/L)	8.2 (4.4)	11.5 (5.5)	0.04 *
AUC0–12 (µg/h/mL)	51.2 (19.8)	53.8 (24.6)	0.72
Dose-normalized AUC (mg/h/L) †	90.1 (45.5)	110.0 (58.6)	0.25

Values represent the mean (standard deviation) or *n* (%). Abbreviations: DM, patients with diabetes mellitus; non-DM, patients without diabetes; Tmax, time to maximum concentration; Cmax, maximum concentration; and AUC_0–12_, area under the concentration–time curve from 0 to 12 h. ^†^ Dose-normalized AUC values were normalized to 1000 mg of mycophenolate mofetil. * *p* < 0.05.

## Data Availability

Not applicable.
